# Effects of Extrusion on the Available Energy and Nutrient Digestibility of Soybean Meal and Its Application in Weaned Piglets

**DOI:** 10.3390/ani14233355

**Published:** 2024-11-21

**Authors:** Ge Zhang, Zhiqiang Sun, Tenghao Wang, Ling Liu, Jinbiao Zhao, Zeyu Zhang

**Affiliations:** 1 State Key Laboratory of Animal Nutrition and Feeding, College of Animal Science and Technology, China Agricultural University, Beijing 100193, China; zhangge0557@163.com (G.Z.); sunbeibei5421@163.com (Z.S.); lingliu321@126.com (L.L.); jinbiaozhao@cau.edu.cn (J.Z.); 2 Zhejiang Qinglian Food Co., Ltd., Jiaxing 314399, China; wangth85@126.com

**Keywords:** soybean meal, extrusion, digestibility, growth performance, digestible energy

## Abstract

Experiments were conducted to investigate the effects of extrusion devices on the available energy and nutrient digestibility of soybean meal (SBM), and further to investigate the impact of different levels of extruded SBM on the growth performance and nutrient digestibility of weaned piglets. In Exp. 1, eighteen crossbreed growing pigs with an initial body weight (BW) of 32.6 ± 2.7 kg were randomly assigned to three treatments, including a corn basal diet, SBM, and extruded SBM diets. In addition, six cannulated pigs (initial BW, 22.3 ± 1.8 kg) were allocated across an 6 × 3 Youden square design. The experimental treatments included a N-free diet and three diets containing 40% of different SBM sources. The results indicated that extrusion reduced the anti-nutritional factors in soybean meal without affecting the digestibility of available energy and nutrients. In Exp. 2, 192 pigs (initial BW, 6.60 ± 0.54 kg) were allocated across four diets supplemented with 0%, 3%, 6%, and 9% extruded SBM. The four treatments were randomized and each comprised six replicate pens containing four boars and four gilts. The results showed that average daily feed intake (ADFI) decreased linearly with the increase in extruded SBM levels from d 0–14 (*p* < 0.05), and average daily gain (ADG) increased; ATTD was higher in the GE, DM, OM, and CP of piglets in the 9% extruded SBM group (*p* < 0.05). In conclusion, the extrusion treatment reduced ANFs in SBM, and adding 9% extruded SBM to the diet improved the growth performance of weaned piglets by increasing the digestibility of nutrients.

## Simple Summary

Soybean meal, as a high-quality protein source, is extensively used in animal diets. However, due to constraints in cost and production, exploring its greater potential and seeking alternative resources constitute a prevalent research focus today. This study evaluates the impact of extrusion processing on soybean meal in terms of digestible energy, metabolizable energy, amino acid digestibility, and anti-nutritional factor content. Furthermore, based on the data from Exp. 1, Exp. 2 investigates the effects of different inclusion levels of extruded soybean meal on the nutrient digestibility and growth performance of piglets.

## 1. Introduction

Protein plays a crucial nutritional role in weaning piglet diets, and soybean meal (SBM) is a high-quality protein source for animal feed due to its rich crude protein (CP) and lysine content [1]. However, SBM contains high levels of anti-nutritional factors (ANFs) that bind to intestinal epithelial cells in mammals, interfering with the digestion and absorption of nutrients [2]. Therefore, it is not recommended to add soybean directly to weanling pigs’ diets [3]. To meet the need for the production of feed ingredients, potential ANFs in SBM are usually inactivated, increasing the digestibility of nutrients through extrusion [4].

Extrusion is a continuous process at high temperatures and short times, which destroys ANFs in SBM, and improves the feeding value [5]. The extrusion of soybeans at 82–137 °C can effectively inactivate ANFs in soybeans [6]. However, twin-screw extrusion may cause a partial loss of CP and amino acids in SBM, as well as a reduction in protein solubility, although it may improve CP digestibility [7]. Extruded feed has been found to increase palatability, which is more conducive to animal digestion and absorption, and greatly helps to improve animal production performance [8]. At present, extruded SBM has been widely used in weaned piglet feed, but few studies have investigated suitable extrusion methods for SBM. Previous studies have shown that extrusion increased the apparent total tract digestibility (ATTD) of dry matter (DM), gross energy (GE), CP, and ether extract (EE) in weaned pigs [6]. Milani et al. [9] reported that the ANFs in SBM could not be well passivated at the extrusion temperature of 120 °C, which negatively affected the average daily gain (ADG) of weaning piglets and finishing pigs. The effect of extrusion may vary for pigs at different stages; feeding extruded SBM diets to growing pigs increased GE and nutrient digestibility, but did not improve growth performance compared to unexpanded SBM diets [10]. Furthermore, feeding weaned piglets an extruded SBM diet may result in a higher feed conversion ratio (FCR) but no significant difference in ADG; it also negatively affects gastric mucosal integrity which increases the risk of peptic ulcers [11]. Hence, it is necessary to evaluate the impact of extrusion on the growth performance of weaned piglets while investigating its effects on raw materials.

The objective of this study was to study the effect of extrusion of SBM on its nutrient composition, energy content, and nutrient digestibility, and further to explore the effect of adding different levels of extruded SBM in the diet on weaning piglets.

## 2. Materials and Methods

### 2.1. Raw Materials and Device Selection

SBM was purchased and used for the further extrusion process (Shennong feed Technology Co., Ltd., Zhengzhou, China). The device selected was a twin-screw extruder with a 200 mm diameter barrel and a length/diameter (L/D) ratio of 20 (Beijing Modern Yanggong Machinery Techology Development Co., Ltd. TPE62S, Beijing, China). The extruder barrel temperature and the screw speed were controlled by a computer, using parameters recommended by the manufacturer for the swine diet. The conditioning temperature was 70–75 °C, the feed section temperature was 75–80 °C, the compression section temperature was 120–125 °C, and the extruder section temperature was 125–130 °C.

### 2.2. Exp. 1: Effects of Extrusion on Energy and Nutrient Digestibility of SBM

This study measured the digestible energy (DE), metabolizable energy (ME), nutrient digestibility, and standardized ileal digestibility (SID) of amino acids (AAs) in SBM for growing pigs before and after extrusion. A total of 18 growing pigs with an initial body weight (BW) of 34.6 ± 2.75 kg were randomly assigned to 3 dietary treatments (N-free diet, SBM diet, and ESBM diet) using a randomized complete block design, with 6 pigs per group. All pigs were housed individually in stainless steel metabolism cages (1.4 m × 0.7 m × 0.6 m), which equipped with a slatted floor, a nipple drinker, and a feeding trough. This experiment included two periods, with the first 7 days as an acclimation period and the last 5 days as a sample collection. The analyzed composition of the experimental diets is shown in Table 1 and Table 2. All diets were supplemented with vitamins and minerals to meet or exceed the nutritional requirements of growing pigs [12].

During the sampling period, fecal samples were collected and stored at −20 °C. To minimize the loss of nitrogen in urine, 25 mL of 6 mol/L HCl was added to the sample bucket (it should be ensured that 10 mL of hydrochloric acid is present in every 1000 mL of urine collected). Each day, urine was filtered through gauze, and 1% of this filtered urine was collected and stored immediately at −20 °C. Both the spilled and remaining feed were collected daily, dried, and weighed. At the end of Exp. 1, the feces were thawed at room temperature, weighed, and recorded. They were then dried at 65 °C for 72 h, and weighed again after 24 h of natural moisture recovery. Additionally, urine samples from each pig were thawed, mixed, and filtered and approximately 40 mL was removed for analysis. The urine and fecal samples were then mixed to correspond to each pig, and stored at −20 °C for subsequent tests.

### 2.3. Exp. 2: Testing with Cannulated Animals in the Ileum

Six barrows, initially weighing 22.3 ± 1.8 kg, were fitted with a T-cannula at the distal ileum in an 6 × 3 Youden square design and allotted to 3 dietary treatments, with 6 pigs per group. The animal care and surgical methods followed the protocols detailed in a previous study [13], and the T-cannula was used as outlined by Huang et al. [14]. After a two-week recovery period, the pigs were weighed and individually accommodated in stainless steel metabolism cages (1.4 m × 0.7 m × 0.6 m). The diets consisted of a N-free diet and 2 test diets containing 40% SBM (SBM and ESBM). The experiment included two periods, and each period lasted 7 days—5 days for acclimation and the final 2 for digestive substance collection. The nutritive composition of the 3 test diets is shown in Table 3.

Digesta were collected on days 6 and 7 of each period following the method described by Stein et al. [13], with a minor adjustment to smaller collection bags (200 mL), and replaced every 30 min. Samples of ileal digesta were collected at 08:00 and 17:00 h of each day, and immediately stored at −20 °C. The digesta samples were thawed and mixed to correspond to each pig and diet, and lyophilized in a vacuum-freeze dryer (Tofflon Freezing Drying Systems, Shanghai, China) for further analysis.

### 2.4. Exp. 3: Effects of Extruded SBM Inclusion Level on Growth Performance and Nutrient Digestibility in Weaned Piglets

The study lasted for 28 days, and 192 healthy weaned pigs (Duroc × Landrace × Yorkshire) with an initial BW of 8.22 ± 0.17 kg were randomly assigned to four dietary groups, with 6 pens per group and 8 pigs (4 barrows and 4 gilts) housed in each pen. Vitamins and minerals were added to meet or exceed the nutritional requirements of weaned pigs [12]. The extruded SBM used in this experiment was consistent with that used in Exp. 1. The addition levels of extruded SBM in the four diets of the weaned piglets were 0%, 3%, 6%, and 9%, respectively. All piglets were housed in 1.5 × 1.5 m^2^ pens which were equipped with plastic-slatted floors, automatic stainless steel nipple drinkers, and feeders. Environmental parameters in the house were controlled by the farm and kept constant. The ingredient compositions and nutrient levels of the experimental diets are shown in Table 4.

The feeding trial was divided into two periods: period 1 lasted from d 0 to d 14, and period 2 from d 15 to d 28. Individual body weights were recorded for each piglet, and feed intakes were recorded for each pen at each period. ADG was calculated for each piglet; the average daily feed intake (ADFI), feed conversion ratio (FCR), and feed-to-gain ratio (F:G) were calculated in pen units; and the diarrhea of each piglet was recorded every day [15]. Fecal samples for digestibility determination were collected in pen units on the last 2–3 days of the second period, followed by drying, the same as in Exp. 1. On days 13 and 27 of the experiment, anterior vena cava blood was collected from boars with near-average body weight in each pen using a vacuum blood collection tube without anticoagulant (Greiner Bio-One GmbH, Frickenhausen, Austria).

### 2.5. Chemical Analysis

In Exp. 1 and Exp. 2, SBM, ESBM, diets, and fecal samples were ground to pass through a 1 mm (40 mesh) screen. GE was determined using bomb calorimetry (Model 6400; Parr Instruments, Moline, IL, USA). DM, CP, EE, and OM were analyzed according to AOAC [16], using filter bags (Model F57, Ankom Technology, Macedonia, NY, USA), and a fiber analyzer (ANKOM200 Fiber Analyzer, Ankom Technology, Macedon, NY, USA) analyzed NDF and ADF [17]. The insoluble dietary fiber (IDF) and soluble dietary fiber (SDF) contents of SBM and ESBM were measured using a Dietary Fiber Analyzer (AnkomTDF Dietary Fiber Analyzer Ankom Technology, Macedon, NY, USA) following AOAC [16]. The AA contents in SBM, ESBM, feed, and digesta were analyzed according to the method described by AOAC [16].ELISA test kits (Beijing Longkefangzhou Bioengineering Technology Co., Ltd., Beijing, China) were used to determine the ANFs of SBM, including glycinin, β-conglycinin, trypsin inhibitor (TI), and lectins.

In Exp. 3, the acid-insoluble ash (AIA) in diets and feces was determined as an endogenous indicator according to the method described by ISO [18]. Other indicators were measured using methods similar to Exp. 1.

### 2.6. Computational Methods

OM, TDF, DE, ME, the ATTD of GE, DM, OM, and CP, and the NDF and ADF of diets were calculated following previous reported methods [19]. The digestibility of CP and AA for both SBM and ESBM was calculated using the formula described by Stein et al. [20]. In Exp. 3, the nutrient ATTD was determined by AIA concentrations in experimental diets and feces using the formula described by Liu et al. [21].

### 2.7. Statistical Analysis

The UNIVERIATE procedure in SAS 9.2 [22] was used to identify data outliers, and no outliers were found in the study. The GLM procedure was employed to analyze variance. The LSMEANS statement was applied to calculate the means of the treatment groups, Tukey’s test was used for multiple comparisons. For Exp. 1 and Exp. 2, each pig was analyzed as an experimental unit, with dietary treatment groups considered as fixed effects. In Exp. 3, each pen was used as the experimental unit to measure growth performance, the incidence of diarrhea, and nutrient digestibility. A contrast statement was used for linear and quadratic trend analyses, and Tukey’s test was employed for multiple comparisons. Significant differences were accepted if *p* < 0.05, and 0.05 ≤ *p* < 0.10 was considered a tendency of change. Values are expressed as least squares means and standard error of the mean (SEM).

## 3. Results

### 3.1. Chemical Composition, Energy Content, and Nutrient Digestibility

#### 3.1.1. Chemical Composition

Table 1 shows the chemical composition of the experimental ingredients. Overall, the nutritional content we have measured is within the reported range [12,23]. The DM content of SBM after extrusion increased by 5.23%. In the present study, the concentrations of glycinin, β-conglycinin, TI, and lectins of SBM were 160.18, 168.19, 8.35, and 0.40 mg/g, respectively, which are higher than previously reported values [24]. Furthermore, after extrusion, the levels of glycinin, β-conglycinin, TI, and lectins in the soybean meal were all reduced.

#### 3.1.2. Energy Content and Nutrient Digestibility

As shown in Table 5, there were no significant differences in DE, ME, and ME/DE of SBM, ranging from 15.78 to 17.16 MJ/kg DM, 14.76 to 16.65 MJ/kg DM, and 0.91 to 0.97, respectively. In our experiment, the DE and ME of extruded SBM were lower than the value reported by Li et al. [25].

As shown in Table 6, there were no significant differences between extruded and unextruded SBM on the apparent ileal digestibility (AID) and SID of CP and AA. Overall, the AID and SID of CP and most of the AAs in SBM were within previously reported values, higher than the values reported by Han et al. and Rodriguez et al. [26,27], and lower than those by NRC, Berrocoso et al., and Liu et al. [12,28,29].

### 3.2. Growth Performance, Diarrhea Incidence, and Nutrient Digestibility of Piglets

#### 3.2.1. Growth Performance and Diarrhea Incidence

As shown in Table 7, the supplementation level of extruded SBM in the diet had no significant effect on the diarrhea rate of weaned piglets. From d 0 to 14 of the experiment, there was no significant difference in the ADG of weaned piglets with different supplementation levels of extruded SBM, but the ADFI of weaned piglets decreased linearly with the increase in extruded SBM (*p* < 0.05). On d 15–28 of the experiment, when the extruded SBM level was 9%, the ADG of weaned piglets was significantly higher than that in the treatment group without extruded SBM (*p* < 0.05). On d 0–28, the ADG of weaned piglets increased linearly with the addition of extruded SBM (*p* < 0.05). No differences in diarrhea incidence were observed between treatment groups during the study.

#### 3.2.2. Nutrient Digestibility

As shown in Table 8, there was no significant difference in nutrient digestibility among piglets with different levels of soybean meal. On d 15–28, when the dietary extruded SBM substitution level was 9%, the ATTD of GE, DM, OM, and CP was significantly higher than that of other treatment groups (*p* < 0.05). The ATTD of GE, DM, OM, and CP increased linearly with increasing dietary extruded SBM levels.

## 4. Discussion

### 4.1. Effects of Extrusion on Chemical Composition and ANF of SBM

Amornthewaphat et al. [30] reported that the crude fiber content decreased after extrusion, which echoed our research, where the content of NDF and ADF decreased by 6.22% and 2.77%, respectively, after extrusion. In this study, extrusion has less effect on the content of CP and EE in SBM. Previous research indicates that the degree of amino acid loss during the extrusion process is associated with extrusion parameters and the variety of SBM [31].

Previous studies have shown that trypsin inhibitors (TIs) decrease the secretion of proteolytic pancreatic enzymes trypsin and chymotrypsin by forming a stable inactive complex, thereby reducing the digestibility of AAs [32]. Different SBM production processes may be one of the factors affecting the ANF content in SBM [33]. The TI in soybean is heat-sensitive, so it can be inactivated by heat treatment [34]. Extrusion removes ANFs and increases nutritional value in soybean at a lower cost than other heating systems [4]. ANFs and protein solubility will decrease with the prolongation of heating time, but excessive heating will affect the soluble protein content, resulting in a decrease in protein digestibility [35]. Karr-Lilienthal et al. [36] found that the protease inhibitor in SBM can still maintain good activity when the extrusion temperature is less than 135 °C. In other crops, such as canola meal, the glucosinolate will decrease at 100 °C extrusion [37]. Pelleting at 75–85 °C was not sufficient to deactivate ANFs, but extrusion at 115 °C deactivated them [38]. We observed that the TI values in extruded SBM were lower than SBM, indicating that the temperature during the extrusion process contributed to the decrease in TI content in SBM. Additionally, the contents of glycinin, β-conglycinin, TI, and lectin all decreased, thereby demonstrating the efficacy of the extrusion treatment on SBM.

### 4.2. Effects of Extrusion on Energy Content and Digestibility of SBM

Among plant protein sources, SBM has high CP and reasonable AA composition, which is an important reason why it is widely used in pig diets [39]. Extrusion disrupts the cell wall of the grain, thereby increasing the ATTD of GE and EE [40]. In this study, there was no marked change in the DE and ME of SBM. From a material point of view, we speculate that the low starch content of the material may affect the extrusion effect [27], and it may be related to the insufficient viscosity of SBM, which reduces the energy input to the material by the machine [41]. In addition, our previous work showed that twin-screw extrusion significantly improved the DE, ME, and ATTD of GE in full-fat rice bran and DDGS [42], but no differences were observed under the test conditions. We hypothesize that this may be due to two factors: firstly, the starch content in the raw materials is different [12], and secondly, the extrusion parameters may require optimization.

Notably, the SID of CP of unextruded SBM was 88.31%, while the SID of CP after extrusion using a twin-screw extruder increased to 90.34%. Heat treatment brings thermal damage to the protein and reduces the concentration of lysine [43]. However, the loss of amino acids in extruded SBM during the experiment was minimal, indicating that twin-screw wet extrusion may be better able to avoid this problem. Similarly, Rodriguez et al. [27] reported that extrusion increases the AID of starch in grains and the SID of most AAs except Lys and Pro. Among the AAs, we observed that the SID of lysine, methionine, threonine, and tryptophan increased after SBM was extruded. Generally speaking, the three-dimensional structure of proteins can often be unfolded by heating, thereby increasing AA digestibility [44]. In this experiment, the digestibility of most AAs increased by 5–10 percentage points after extrusion treatment, which makes it possible to reduce the addition of SBM or other protein materials to the diet.

### 4.3. Effects of Different Substitution Ratios of Extruded SBM on Growth Performance, Nutrient Digestibility, and Diarrhea Incidence of Piglets

Extrusion increases the ratio of rapidly digestible starch and decreases the ratio of slowly digestible starch and resistant starch to alter piglet gut microbiota and biological processes, thereby reducing the prevalence of swine dysentery [45]. However, no differences in diarrhea incidence between the four groups were observed in the study, which may be because the RS content of SBM itself is low, and other influencing factors need to be considered. Overall, the addition of 9% extruded SBM to the diet had no significant effect on piglet diarrhea incidence. In this experiment, the ADG of weaned piglets in the diet treatment group with 9% extruded SBM supplementation level on d 14–28 was significantly higher than that in the diet treatment group without extruded SBM. The high pressure and temperature of extrusion increases starch gelatinization, thereby improving nutrient palatability, digestibility, and growth performance [46]. However, there was no significant difference in feed intake among the four groups in this experiment, and the ADFI of weaned piglets decreased linearly with the increase in the proportion of extruded SBM. The results of this study showed that the ADG of weaned piglets in the 9% extruded SBM group was significantly higher than that in the SBM group on d 14–28. Similarly, Lundblad et al. [47] reported that extruder processing significantly increased ADG and the gain-to-feed ratio (G:F) in weaned piglets on d 0–36. Therefore, the addition of extruded SBM to the diet may improve post-weaning ADG and G:F by providing more energy.

Extruded soybean at 122 °C significantly improved the ATTD of GE, CP, and EE by reducing TI [6]. Similarly to our results, we observed the highest digestibility of GE, DM, OM, and CP in the 9% extruded SBM diets group, which may explain why the ADG of weaned piglets in the 9% extruded SBM group was higher than that in the control group on d 14–28. Friesen et al. [48] found that the growth performance of extruded SBM had a promoting effect on the later growth of piglets. We observed similar results, namely, that the different substitution of extruded SBM to the diet had no effect on the ATTD in the early stage (d 0–14), and the effect mainly existed in the later stage of weaning (d 15–28). Zhuo et al. [49] reported that the ATTD of DM and GE increased when corn and rice were extruded, and we noticed that these two cereals have higher starch, which is the main target of extrusion. It is, therefore, possible that the addition of extruded SBM to the diet may improve the growth performance of weaned piglets by increasing nutrient digestibility.

## 5. Conclusions

In summary, the extrusion process reduces glycinin and β-conglycinin in SBM, but it does not significantly affect the ATTD of nutrients and AAs in growing pigs. At the same time, the addition of 9% extruded SBM into the feed can enhance the growth performance of weaned piglets by improving the digestibility of nutrients.

## Figures and Tables

**Table 1:** Chemical composition of the experimental ingredients ^1^ (%, DM basis).

Items	SBM	ESBM
GE, MJ/kg	20.35	19.75
DM	87.4	92.63
OM	93.28	98.33
CP	49.50	49.21
EE	1.25	1.35
NDF	21.48	15.26
ADF	11.20	8.43
TDF	23.23	24.08
SDF	2.23	2.75
IDF	21.00	21.34
Ash	6.72	7.65
Glycinin (mg/g)	160.18	40.05
β-conglycinin (mg/g)	168.19	51.49
Trypsin inhibitor (mg/g)	8.35	7.32
Lectins (mg/g)	0.40	<0.01
Indispensable AA		
Arg	3.51	3.54
His	1.32	1.30
Leu	3.94	3.96
Ile	2.43	2.45
Lys	3.30	3.30
Met	0.66	0.65
Phe	0.71	0.73
Thr	2.01	2.01
Trp	0.78	0.76
Val	2.54	2.57
Dispensable AA		
Ala	2.24	2.27
Asp	5.80	5.81
Cys	2.56	2.55
Glu	9.32	9.36
Gly	2.16	2.19
Pro	2.65	2.63
Ser	2.53	2.55

^1^ SBM: soybean meal; ESBM: extruded soybean meal; GE, gross energy; DM, dry matter; OM, organic matter; CP, crude protein; EE, ether extract; NDF, neutral detergent fiber; ADF, acid detergent fiber.

**Table 2:** Ingredient composition and nutrient levels of the digestible and metabolizable experimental diets ^1^ (%, as-fed basis).

Items	Basal Diet	SBM	ESBM
Ingredients			
Corn	96.90	67.83	67.83
SBM	-	29.07	-
Extruded SBM	-	-	29.07
Dicalcium phosphate	1.70	1.70	1.70
Limestone	0.60	0.60	0.60
NaCl	0.30	0.30	0.30
Premix ^2^	0.50	0.50	0.50
Analyzed nutrient levels			
GE, MJ/kg	16.28	16.51	15.92
DM	89.41	89.48	87.47
OM	85.94	86.27	82.72
CP	8.55	19.08	18.82
EE	3.05	2.56	2.98
NDF	9.39	12.14	9.75
ADF	2.38	4.13	3.23
Ash	3.46	3.21	4.75

^1^ SBM: soybean meal; ESBM: extruded soybean meal; GE, gross energy; DM, dry matter; OM, organic matter; CP, crude protein; EE, ether extract; NDF, neutral detergent fiber; ADF, acid detergent fiber. ^2^ Premix provided the following per kilogram of feed: vitamin A, 12,000 IU as vitamin A acetate; vitamin D, 2500 IU as vitamin D3; vitamin E, 30 IU as DL-α-tocopheryl acetate; vitamin B12, 12 μg; vitamin K, 3 mg as menadione sodium bisulfate; D-pantothenic acid, 15 mg as calcium pantothenate; nicotinic acid, 40 mg; choline, 400 mg as choline chloride; Mn, 30 mg as manganese oxide; Fe, 90 mg as iron sulfate; Cu, 10 mg as copper sulfate; I, 0.35 mg as ethylenediamine dihydroiodide; and Se, 0.3 mg as sodium selenite.

**Table 3:** Ingredient composition and nutrient levels of the AA digestibility experimental diets ^1^ (%, as-fed basis).

Items	N-Free Diet	SBM	ESBM ^1^
Ingredients			
Corn starch	68.90	34.40	34.40
SBM	-	40.00	-
ESBM	-	-	40.00
Sucrose	20.00	20.00	20.00
Cellulose acetate	4.00	-	-
Soybean oil	3.00	3.00	3.00
Dicalcium phosphate	1.60	1.00	1.00
Limestone	1.00	0.50	0.50
Potassium carbonate	0.30	-	-
Magnesium oxide	0.10	-	-
NaCl	0.30	0.30	0.30
Chromic oxide	0.30	0.30	0.30
Premix ^2^	0.50	0.50	0.50
Total	100.00	100.00	100.00
Analyzed nutrient levels			
CP	0.96	17.07	16.80
Indispensable AAs			
Arg	0.01	2.08	2.16
His	0.01	0.73	0.75
Leu	0.02	0.92	0.95
Ile	0.02	3.37	3.48
Lys	0.01	0.95	1.02
Met	0.02	0.77	0.81
Phe	0.01	0.54	0.59
Thr	0.02	0.22	0.24
Trp	0.01	0.93	0.95
Val	0.02	0.20	0.20
Dispensable AAs			
Ala	0.03	0.87	0.90
Asp	0.02	1.38	1.42
Cys	0.01	0.80	0.83
Glu	0.04	0.91	0.94
Gly	0.01	0.47	0.48
Pro	0.05	1.14	1.18
Ser	0.01	1.29	1.34
Tyr	0.02	0.27	0.24
Ala	0.03	0.87	0.90

^1^ SBM: soybean meal; ESBM: extruded soybean meal. ^2^ Premix provided the following per kilogram of feed: vitamin A, 12,000 IU as vitamin A acetate; vitamin D, 2500 IU as vitamin D3; vitamin E, 30 IU as DL-α-tocopheryl acetate; vitamin B12, 12 μg; vitamin K, 3 mg as menadione sodium bisulfate; D-pantothenic acid, 15 mg as calcium pantothenate; nicotinic acid, 40 mg; choline, 400 mg as choline chloride; Mn, 30 mg as manganese oxide; Fe, 90 mg as iron sulfate; Cu, 10 mg as copper sulfate; I, 0.35 mg as ethylenediamine dihydroiodide; and Se, 0.3 mg as sodium selenite.

**Table 4:** Ingredient composition and nutrient levels of the experimental diets ^1^ (%, as-fed basis).

Items	ESBM (%)			
	0	3	6	9
Ingredients				
Corn	65.38	65.32	65.30	65.24
Extruded soybean meal	-	3.00	6.00	9.00
Soybean meal	9.00	6.00	3.00	-
Soy protein concentrate	6.00	6.00	6.00	6.00
Extruded full-fat soybean	5.00	5.00	5.00	5.00
Fish meal	2.95	3.00	3.00	3.05
Whey powder	6.00	6.00	6.00	6.00
Soybean oil	2.20	2.20	2.20	2.20
Dicalcium phosphate	1.00	1.00	1.00	1.00
Limestone	0.80	0.80	0.80	0.80
NaCl	0.20	0.20	0.20	0.20
L-Lysine-HCl	0.60	0.61	0.62	0.62
DL-Methionine	0.10	0.10	0.10	0.10
L-Threonine	0.22	0.22	0.22	0.22
L-Tryptophan	0.05	0.05	0.06	0.07
Premix ^2^	0.50	0.50	0.50	0.50
Calculated nutrient levels, %				
DE, MJ/kg	14.36	14.36	14.36	14.37
CP	18.57	18.59	18.57	18.55
SID Lysine	1.35	1.35	1.35	1.34
SID Methionine	0.41	0.41	0.40	0.40

^1^ Extruded soybean meal. ^2^ Premix provided the following per kilogram of feed: vitamin A, 12,000 IU as vitamin A acetate; vitamin D, 2500 IU as vitamin D3; vitamin E, 30 IU as DL-α-tocopheryl acetate; vitamin B12, 12 μg; vitamin K, 3 mg as menadione sodium bisulfate; D-pantothenic acid, 15 mg as calcium pantothenate; nicotinic acid, 40 mg; choline, 400 mg as choline chloride; Mn, 30 mg as manganese oxide; Fe, 90 mg as iron sulfate; Cu, 10 mg as copper sulfate; I, 0.35 mg as ethylenediamine dihydroiodide; and Se, 0.3 mg as sodium selenite.

**Table 5:** Effect of extrusion on the available energy and digestibility of soybean meal ^1^ (%, DM basis).

Items	SBM	ESBM	SEM	*p* Value
Energy content, MJ/kg				
DE	16.43	16.67	0.25	0.66
ME	15.60	16.19	0.28	0.32
ME/DE	0.95	0.97	0.01	0.19
ATTD, %				
GE	81.62	83.35	2.10	0.70
DM	80.94	81.30	1.64	0.92
OM	82.15	83.22	1.56	0.75
CP	87.30	88.19	1.04	0.69
EE	70.38	74.36	1.99	0.36
NDF	70.61	75.40	2.68	0.22
ADF	63.24	69.87	2.59	0.94

^1^. SBM: soybean meal; ESBM: extruded soybean meal; SEM, standard error of the mean; DE, digestible energy; ME, metabolizable energy; GE, gross energy; DM, dry matter; OM, organic matter; CP, crude protein; EE, ether extract; NDF, neutral detergent fiber; ADF, acid detergent fiber. *n* = 6.

**Table 6:** Effect of extrusion on the apparent and standardized ileal digestibility of crude protein and amino acids of soybean meal ^1,2^ (%).

Items	AID				SID			
	SBM	ESBM	SEM	*p* Value	SBM	ESBM	SEM	*p* Value
CP	78.5	80.17	1.89	0.72	88.31	90.37	1.89	0.71
Indispensable AAs								
Arg	89.22	90.03	1.6	0.48	92.77	96.51	1.6	0.3
His	84.75	86.96	2.05	0.42	88.9	93.64	2.05	0.28
Leu	78.4	86.71	3.25	0.17	83.28	92.9	3.25	0.14
Ile	80.77	87.48	2.85	0.18	85	92.91	2.85	0.15
Lys	80.05	84.87	3.05	0.37	84.31	90.33	3.05	0.33
Met	78.47	91.15	3.78	0.12	83.77	94.66	3.78	0.19
Phe	80.56	86.66	2.73	0.19	78.16	88.62	3.86	0.24
Thr	72.71	79.81	3.71	0.29	82.2	92.19	3.71	0.2
Trp	82.16	83.63	2.83	0.94	88.04	91.5	2.83	0.72
Val	76.96	84.26	3.43	0.26	82.67	92.28	3.43	0.18
Dispensable AAs								
Ala	68.86	76.6	4.86	0.39	77.76	87.92	4.86	0.33
Asp	81.92	83.74	2.05	0.31	85.86	89.33	2.05	0.31
Cys	68.53	77.32	3.86	0.34	84.56	92.17	2.73	0.15
Glu	85.07	87.79	2.18	0.63	87.81	92.46	2.18	0.39
Gly	70.95	68.06	3.51	0.88	87.26	89.43	3.51	0.74
Ser	79.46	83.05	2.46	0.29	86.17	91.92	2.46	0.23
Tyr	78.6	87.16	3.57	0.2	83.13	93.17	3.57	0.17

^1^ AID: apparent ileal digestibility; SID: standardized ileal digestibility; ESBM: extruded soybean meal; SEM, standard error of the mean. *n* = 6. ^2^. Values for SID are calculated by correcting the apparent ileal digestibility (AID) values with the basal endogenous losses. Basal ileal endogenous losses were determined (g/kg of dry matter intake) as crude protein, 17.36; Arginine, 0.46; Histidine, 0.19; Isoleucine, 0.37; Leucine, 0.67; Lysine, 0.48; Methionine, 0.69; Phenylalanine, 0.37; Threonine, 0.92; Tryptophan, 0.16; Valine, 0.11; Alanine, 0.53; Aspartic acid, 0.61; Cysteine, 0.82; Glutamic acid, 1.26; Glycine, 0.21; Proline, 0.71; Serine, 3.22; Tyrosine, 0.24.

**Table 7:** Effect of different extruded SBM level on growth performance and diarrhea rate of weaned pigs.

	Extruded SBM (%)				SEM	*p* Value		
Items	0	3	6	9		Treatment	Linear	Quadratic
Initial BW, kg	8.25	8.23	8.22	8.2	0.17	0.99	0.93	0.99
Medial BW, kg	11.32	11.42	11.29	11.05	0.22	0.95	0.65	0.72
Final BW, kg	17.43	17.83	17.77	18.00	0.30	0.93	0.57	0.90
Days 0 to 14								
ADG, g/d	220	218	220	203	16.05	0.85	0.49	0.64
ADFI, g/d	374	354	354	332	11.81	0.12	0.02	0.94
G:F	0.60	0.65	0.62	0.61	0.02	0.38	0.96	0.35
Diarrhea rate %	4.65	3.87	4.32	4.46	0.84	0.94	0.98	0.62
Days 14 to 28								
ADG, g/d	436 ^b^	468 ^ab^	462 ^ab^	497 ^a^	12.25	0.02	0.32	0.34
ADFI, g/d	741	755	740	799	34.60	0.65	0.92	0.56
G:F	0.57	0.60	0.60	0.60	0.01	0.17	0.13	0.17
Diarrhea rate %	3.81	2.86	3.57	2.68	0.55	0.53	0.48	0.74
Days 0 to 28								
ADG, g/d	309	320	314	343	12.61	0.27	0.04	0.29
ADFI, g/d	517	494	514	542	22.16	0.53	0.35	0.32
G:F	0.58	0.62	0.61	0.60	0.01	0.58	0.52	0.26
Diarrhea rate %	4.17	2.98	3.27	3.21	0.56	0.49	0.34	0.34

BW, body weight; G:F, Gain-to-feed ratio. ^a–b^ different superscript in the same column means significantly different. *n* = 6.

**Table 8:** Effect of different extruded SBM level on nutrient digestibility in weaned pigs.

Items	Extruded SBM (%)				SEM	*p* Value		
	0	3	6	9		Treatment	Linear	Quadratic
Day 14								
ATTD, %								
GE	90.26	90.81	90.67	91.48	0.48	0.39	0.13	0.79
DM	90.50	91.22	91.02	91.77	0.43	0.29	0.09	0.98
OM	91.67	92.31	92.12	92.77	0.40	0.35	0.12	0.99
CP	84.53	85.50	86.01	87.09	0.92	0.32	0.07	0.95
EE	80.95	82.97	82.00	83.55	1.14	0.45	0.21	0.84
NDF	72.68	73.96	73.04	75.31	1.18	0.44	0.22	0.69
ADF	71.54	73.32	72.65	71.12	1.32	0.64	0.56	0.24
Day 28								
ATTD, %								
GE	89.55 ^b^	89.90 ^b^	89.71 ^b^	91.91 ^a^	0.49	<0.01	<0.01	0.07
DM	89.83 ^b^	90.32 ^b^	90.07 ^b^	91.18 ^a^	0.45	<0.01	<0.01	0.09
OM	91.10 ^b^	91.53 ^b^	91.32 ^b^	93.31 ^a^	0.44	<0.01	<0.01	0.09
CP	82.94 ^b^	84.45 ^b^	84.46 ^b^	88.16 ^a^	0.83	<0.01	<0.01	0.15
EE	80.76	82.37	81.64	85.25	1.35	0.14	0.05	0.47
NDF	71.76 ^ab^	71.45 ^ab^	66.90 ^b^	73.75 ^a^	1.50	0.03	0.83	0.03
ADF	70.83	70.87	69.32	71.65	0.76	0.82	0.91	0.52

ATTD: apparent total tract digestibility; GE, gross energy; DM, dry matter; OM, organic matter; CP, crude protein; EE, ether extract; NDF, neutral detergent fiber; ADF, acid detergent fiber. *n* = 6. ^a–b^ Different superscript within a row means significantly different (*p* < 0.05).

## Data Availability

The original contributions generated for this study are included in the article; further inquiries can be directed to the corresponding author.
